# Systematic Review and Meta-Analysis of Published Studies on Endovascular Repair of Abdominal Aortic Aneurysm With the p-Branch

**DOI:** 10.3389/fsurg.2022.879682

**Published:** 2022-04-29

**Authors:** Haoliang Wu, Liwei Zhang, Mingxing Li, Shunbo Wei, Cong Zhang, Hualong Bai

**Affiliations:** ^1^Department of Vascular and Endovascular Surgery, First Affiliated Hospital of Zhengzhou University, Zhengzhou, China; ^2^Key Vascular Physiology and Applied Research Laboratory of Zhengzhou City, Zhengzhou, China

**Keywords:** p-Branch, off-the-shelf, branched endovascular aortic aneurysm repair, juxtarenal abdominal aortic aneurysm, pararenal abdominal aortic aneurysm

## Abstract

**Background:**

Endovascular treatment of juxtarenal or pararenal abdominal aortic aneurysms is more popular than open surgery, mainly because it reduces perioperative mortality and morbidity. The custom-made fenestrated devices need to be tailored to each patient, so these devices require extra manufacturing and shipping time. The increased wait time may increase the risk of aneurysm rupture in some patients. In some situations, “Off-the-shelf” (OTS) fenestrated grafts can be used. The Cook Zenith p-Branch device (William Cook Australia, Brisbane, Australia) is a relatively common OTS. This study aimed to systematically evaluate all published experiences with p-Branch.

**Methods:**

We searched PubMed, Embase, and Cochrane to find works of literature that reported on the outcomes of patients treated with the p-Branch stent-grafts. Then we conducted an assessment of quality and meta-analysis of the results. The primary endpoints were the application rate of p-Branch stent-graft (type A, B), technical success rate, and early re-intervention rate. We estimated pooled proportions and 95% CIs.

**Results:**

Initial search of the literature included 111 articles, of which 7 studies were included in the end. A total of 260 patients were enrolled in these studies, and 218 patients were eventually treated with p-Branch. The pooled application rate of type A devices was 48% (95% CI, 29–67%), and pooled application rate of type B devices was 30% (95% CI, 16–44%). The pooled technical success rate was 87% (95% CI, 75–98%). The early re-intervention rate was 10% (95% CI, 3–17%). Midterm renal infarct rate (after 30 days) was 3% (95% CI, 0–6%). Midterm re-intervention rate (after 30 days) was 30% (95% CI, 3–57%). Midterm renal failure rate (after 30 days) was 6% (95% CI, 2–10%).

**Conclusions:**

This pooled analysis indicated an acceptable technical success rate after p-Branch stent-graft implantation, with early and midterm re-intervention rate and renal failure rate that cannot be ignored. The p-Branch repair of juxtarenal abdominal aortic aneurysms may be an appropriate and safe option, especially in emergency situations.

## Introduction

Endovascular treatment is often preferred over open surgical aneurysm repair for repairing juxtarenal or pararenal abdominal aortic aneurysms (AAA). Some studies have pointed out that the short- and medium-term mortality and morbidity rates of endovascular treatment were lower (1–3). In addition, unfavorable neck anatomy with insufficient infrarenal sealing zone poses a great challenge for endovascular treatment of AAA. Fenestrated and branched stent-grafts could be used to treat complex aortic aneurysms at high risk (4).

In the past few years, device technology and operator experience in endovascular aortic repair have achieved tremendous improvement, resulting in improved outcomes with fenestrated and branched endografts (5). And current studies suggested fenestrated endovascular aneurysm repair (FEVAR) was a safe and effective treatment for juxtarenal and pararenal AAA (6, 7). These custom-made fenestrated devices, which need to be tailored to each patient, require extra manufacturing and shipping time, making them unavailable to patients requiring emergency interventions. At the same time, this also puts large-diameter aneurysms at a heightened risk of rupture (8, 9).

Physician-modified fenestrated stent-grafts (PMSGs) save the time required for graft manufacture and delivery, but there are still technical challenges and concerns about such uncontrolled device modifications (10–12). “Off-the-shelf” (OTS) fenestrated grafts can be used in emergency situations due to a degree of standardized design in planning and deployment. The emergence of OTS solves the dilemma faced by the above devices to a certain extent. The Cook Zenith p-Branch device (William Cook Australia, Brisbane, Australia) is a relatively common OTS.

The aim of our study was to perform a systematic review and meta-analysis of published reports concerning technical success rate and early and midterm clinical outcomes of the p-Branch stent graft use for the treatment of juxtarenal or pararenal AAA.

## Methods

The present systematic review and meta-analysis was written based on the Preferred Reporting Items for Systematic reviews and Meta-Analysis (PRISMA) Statement (13).

### Eligibility Criteria

This analysis included original research studies that reported outcomes of applications of the p-Branch stent graft for the treatment of juxtarenal or pararenal AAA. The article was considered for inclusion when the target population was patients with aortic aneurysm receiving p-Branch stent-graft treatment and the AAA was objectively diagnosed. Studies examining insufficient data were excluded, as were review articles and studies whose data was incomplete.

### Search Strategy

The databases search was updated last on January 2022 in the PubMed, Embase, and Cochrane Library. No restriction on language was required. Search terms included “p-Branch”, “off-the-shelf”, “aortic aneurysm”, “aneurysm”, and “Zenith”. Moreover, we enriched the search by manually reviewing the reference lists of all retrieved articles.

### Study Selection

Three review authors (HW, LZ, and ML) screened the titles and abstracts of each search result independently. Then we read the full text to review for eligibility and quality of selected articles. Disagreements were resolved by consensus if necessary.

### Data Extraction and Management

Two review authors (SW and CZ) independently extracted data from each study using standard forms. We collected the following data: number, sex, and age of enrolled patients, types of studies, and number of patients. The main endpoints of the analysis were the application rate of the p-Branch stent-graft (type A, B), technical success rate, and early re-intervention rate. Secondary endpoints included midterm renal infarct rate, re-intervention rate, and renal failure (14).

### Assessment of Methodological Quality

The quality of studies was assessed based on The Quality Appraisal of Case Series Studies Checklist (Table 1) (22). We evaluated quality based on it with discrepancies resolved by a third author.

**Table 1:** Quality appraisal checklist for the included studies.

	**Study objective**	**Study design**				**Study population**			**Intervention and cointervention**	
Studies	1. Was the hypothesis/aim/objective of the study clearly stated?	2. Was the study conducted prospectively?	3. Were the cases collected in more than one center?	4. Were patients recruiter consecutively?		5. Were the characteristics of the patients included in the study described?	6. Were the eligibility criteria (i.e., inclusion and exclusion criteria) for entry into the study clearly stated?	7. Did patients enter the study at a similar point in the disease?	8. Was the intervention of interest clearly described?	9. Were additional interventions (cointerventions) clearly described?
Bargay-Juanet al. (15)	Y	N	N	Y		Partial/unclear	Partial/unclear	N	Partial/unclear	N
Sveinsson et al. (16)	Y	Y	N	Y		Y	Y	N	Y	Y
Farber et al. (17)	Y	Y	N	Y		Y	Y	N	Y	Partial/unclear
Farber et al. (18)	Y	Y	Y	Y		Y	Y	N	Y	Y
Farber et al. (19)	Y	Y	Y	Y		Y	Y	Partial/unclear	Y	N
Kitagawa et al. (20)	Y	Partial/unclear	Partial/unclear	Y		Y	Partial/unclear	N	Y	Y
Ou et al. (21)	Y	Y	Partial/unclear	Y		Partial/unclear	Partial/unclear	Partial/unclear	Y	Partial/unclear
**Outcome measures**				**Statistical analysis**	**Results and conclusions**					**Competing interests and sources of support**
10. Were relevant outcome measures established a priori?	11. Were outcome assessors blinded to the intervention that patients received?	12. Were the relevant outcomes measured using appropriate objective/subjective methods?	13. Were the relevant outcome measures made before and after the intervention?	14. Were the statistical tests used to assess the relevant outcomes appropriate?	15. Was follow-up long enough for important events and outcomes to occur?	16. Were losses to follow-up reported?	17. Did the study provide estimates of random variability in the data analysis of relevant outcomes?	18. Were the adverse events reported?	19. Were the conclusions of the study supported by the results?	20. Were both competing interests and sources of support for the study reported?
Partial/unclear	N	Y	Partial/unclear	Y	Y	Partial/unclear	N	Y	Y	Partial/unclear
Y	N	Y	Y	Y	Y	N	N	Y	Y	Y
Y	N	Y	Y	Y	Y	N	Partial/unclear	Y	Y	Y
Y	N	Y	Y	Y	Y	Y	Y	Y	Y	Y
Y	N	Y	Y	Y	Y	N	N	Y	Y	Y
Y	N	Y	Y	Y	Y	Y	Partial/unclear	Y	Y	Y
Y	N	Y	Partial/unclear	Y	Y	Partial/unclear	N	Y	Y	Y

### Statistical Analysis and Data Synthesis

All analyses were conducted using Stata statistical software version 14 (StataCorp LP, College Station, TX, USA). According to the data collected, we generated pooled rates and 95% CIs. The software produced forest plots and the heterogeneity of included studies was evaluated by providing inconsistency (I^2^) statistics. Publication bias was assessed by generating funnel plots.

## Result

### Study Characteristics

**Figure 1:**
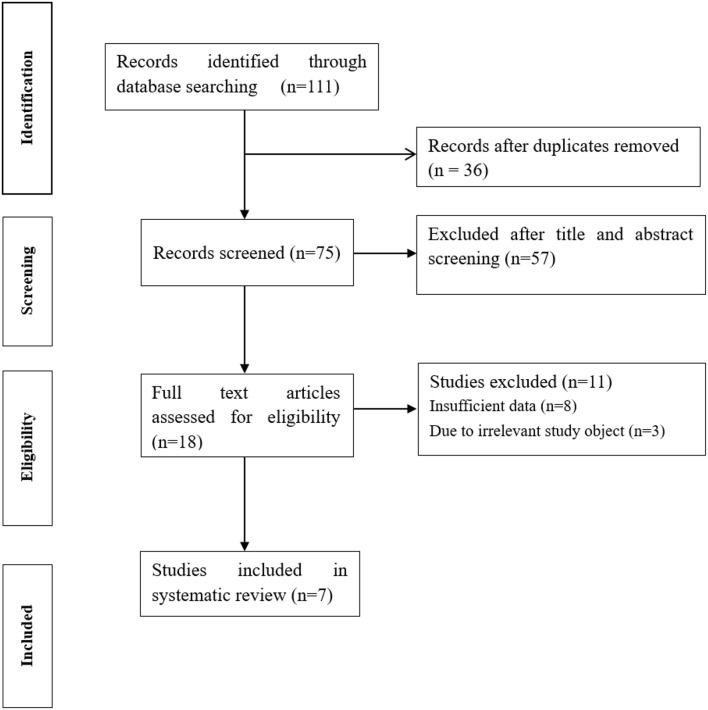
Flow chart of the study selection process.

A total of 111 results were retrieved from databases. After excluding 36 duplicated studies, the remaining 75 studies were potentially eligible. After scanning titles and abstracts and removing 57 irrelevant studies, 18 studies were further evaluated. After reading the full text, 7 eligible studies were finally included, (15–21) 8 studies were excluded due to insufficient data, and 3 studies were excluded due to irrelevant study objects (Figure 1).

The baseline characteristics of the 7 eligible studies included in the present review are shown in Tables 2, 3. A total of 260 patients were enrolled in these studies, and 218 patients were eventually treated with p-Branch. The mean age was 73.1 years; 180 of 219 (82.2%) were men (results from 6 studies). One included publication was a retrospective single-center study, five publications were prospective studies, and one did not specify the nature of the study. Three studies identified a total of 24 patients who received emergency procedures, seventeen of the patients underwent emergent surgery for ruptured aneurysms, three had symptoms, one had a mycotic aneurysm, and the rest were with no information available. In these studies, no death at 30-days was mentioned. The technical success rate was 91.7% (22/24) in the emergent group and 92.6% (162/175) in the elective group.

**Table 2:** Baseline characteristics of studies not funded by Cook.

**Study**	**Country**	**Study type**	**Study period**	**Type of stent**	**No. of patients**		**No. of patients who received a p-Branch**		**Emergent procedure**
Bargay-Juanet al. (15)	Espana	Retrospective, single center	2008–2015	Zenith	41		30		NA
Sveinsson et al. (16)	Denmark	Prospective, single center	2012–2015	Zenith	23		23		11
Farber et al. (18)	USA, Europe	Prospective, multiple center	2011–2015	Zenith	76		76		11
Ou et al. (21)	China	Prospectively	2006–2013	Zenith	51		31		NA
**Study**	**Mean age, years**	**Male, (%)**	**CAD, (%)**	**Smokers, (%)**	**Congestive heart failure**	**MI**	**Hypertension**	**COPD, (%)**	**CKD, (%)**
Bargay-Juanet al. (15)	NA	NA	NA	NA	NA	NA	NA	NA	NA
Sveinsson et al. (16)	69 (52–81)	18 (78)	6 (26)	19 (83)	1 (4)	4 (17)	14 (61)	6 (26)	3 (13)
Farber et al. (18)	71.8 (52–92)	62 (82)	34 (45)	24 (34)	6 (7.9)	21 (28)	63 (83)	24 (32)	15 (20)
Ou et al. (21)	76.8	43	NA	NA	NA	NA	NA	NA	NA
**Study**	**Diabetes mellitus**	**CVD, (%)**	**Hospital days**	**ICU days**	**Endoleak**	**Funding**			**Follow-up**
Bargay-Juanet al. (15)	NA	NA	NA	NA	NA	NA			NA
Sveinsson et al. (16)	3 (13)	2 (9)	8 (4–57)	1 (0–22)	3II	NA			45 ± 24.4
Farber et al. (18)	8 (11)	10 (13)	1.9 (0–22)	7.5 (1–57)	2I 18II	NA			25 ± 13
Ou et al. (21)	NA	NA	NA	NA	NA	NA			NA

*CAD, coronary artery disease; MI, myocardial infarction; COPD, chronic obstructive pulmonary disease; CKD, chronic kidney disease; CVD, cerebrovascular disease; ICU, intensive care unit*.

**Table 3:** Baseline characteristics of studies funded by Cook.

**Study**	**Country**	**Study type**	**Study period**	**Type of stent**	**No. of patients**		**No. of patients who received a p-Branch**		**Emergent procedure**
Farber et al. (17)	USA	Prospective, single center	2012–2013	Zenith	23		14		NA
Farber et al. (19)	USA	Prospective, multiple center	2013–2015	Zenith	30		28		NA
Kitagawa et al. (20)	NA	NA	2011–2012	Zenith	16		16		2
**Study**	**Mean age, years**	**Male, (%)**	**CAD, (%)**	**Smokers, (%)**	**Congestive heart failure**	**MI**	**Hypertension**	**COPD, (%)**	**CKD, (%)**
Farber et al. (17)	72	14 (87.5)	7 (43.8)	NA	1 (6.3)	4 (25)	13 (81.3)	10 (62.5)	3 (18.7)
Farber et al. (19)	73	28 (93.3)	10 (33.3)	9 (30)	5 (16.7)	6 (20)	27 (90)	11 (36.7)	2 (6.7)
Kitagawa et al. (20)	75 (59–87)	15 (94)	13 (81)	14 (88)	NA	9 (56)	15 (94)	6 (38)	1 (6)
**Study**	**Diabetes mellitus**	**CVD, (%)**	**Hospital days**	**ICU days**	**Endoleak**	**Funding**			**Follow-up**
Farber et al. (17)	NA	NA	NA	NA	0	Cook			6.5
Farber et al. (19)	5 (16.7)	2 (6.6)	NA	NA	NA	Cook			29 ± 12.5
Kitagawa et al. (20)	4 (25)	4 (25)	NA	NA	NA	Cook			4.3

*CAD, coronary artery disease; MI, myocardial infarction; COPD, chronic obstructive pulmonary disease; CKD, chronic kidney disease; CVD, cerebrovascular disease; ICU, intensive care unit*.

### Meta-Analysis

**Figure 2:**
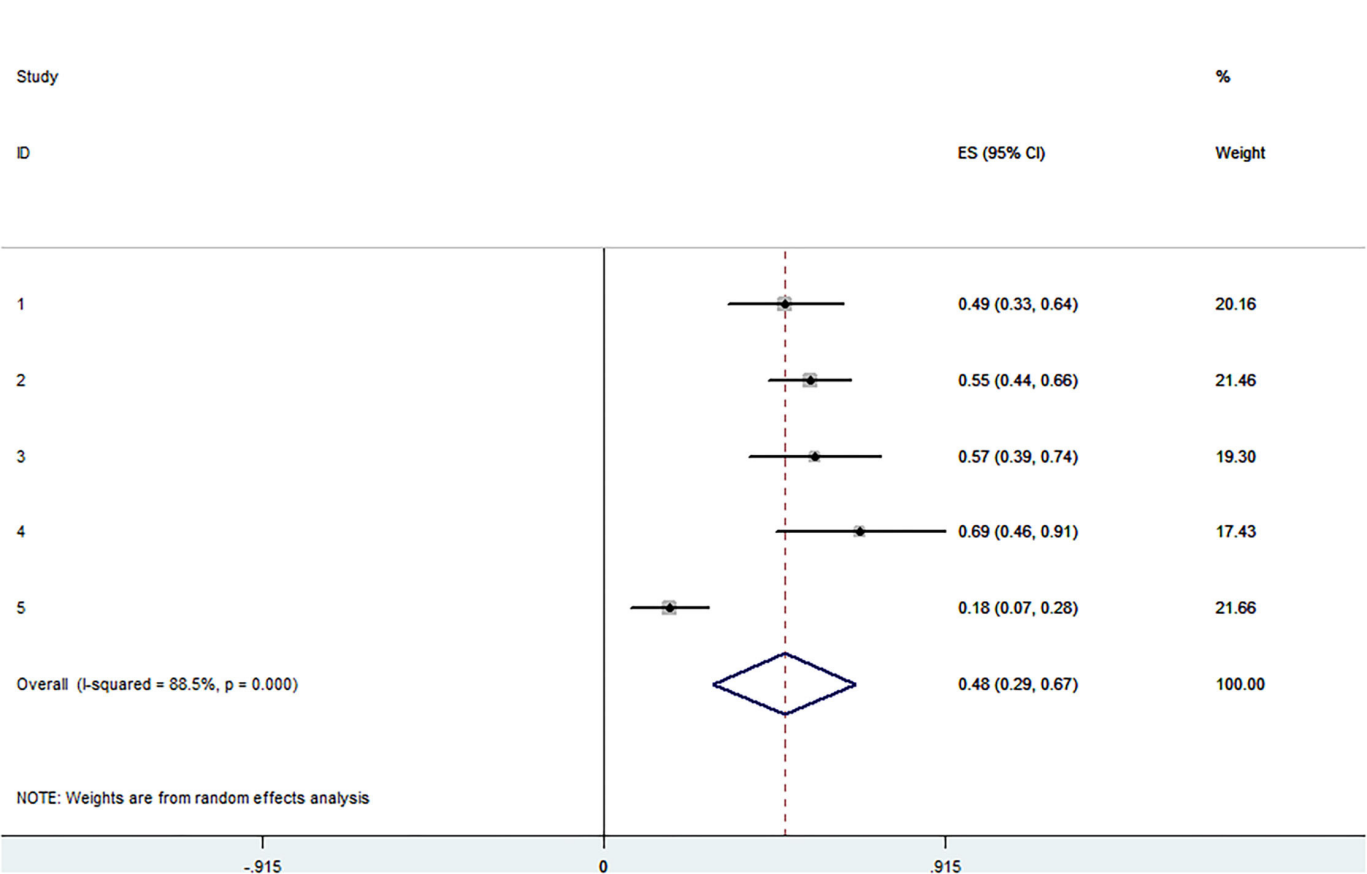
Forest plot presenting the meta-analysis of application rate of type A. CI, Confidence intervals; ES, Effect size.

**Figure 3:**
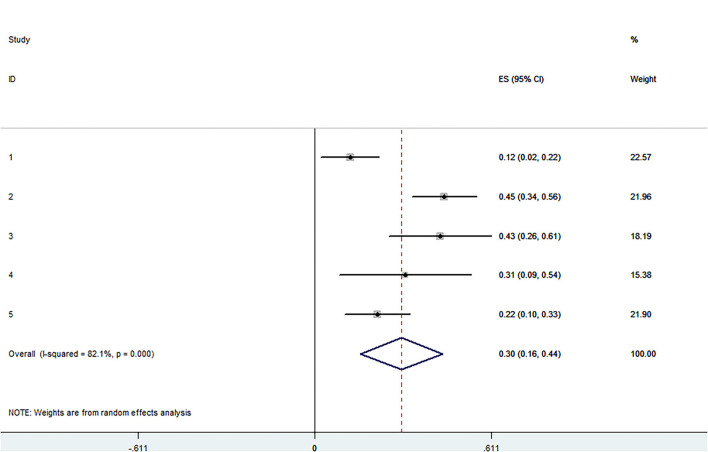
Forest plot presenting the meta-analysis of application rate of type B. CI, Confidence intervals; ES, Effect size.

**Figure 4:**
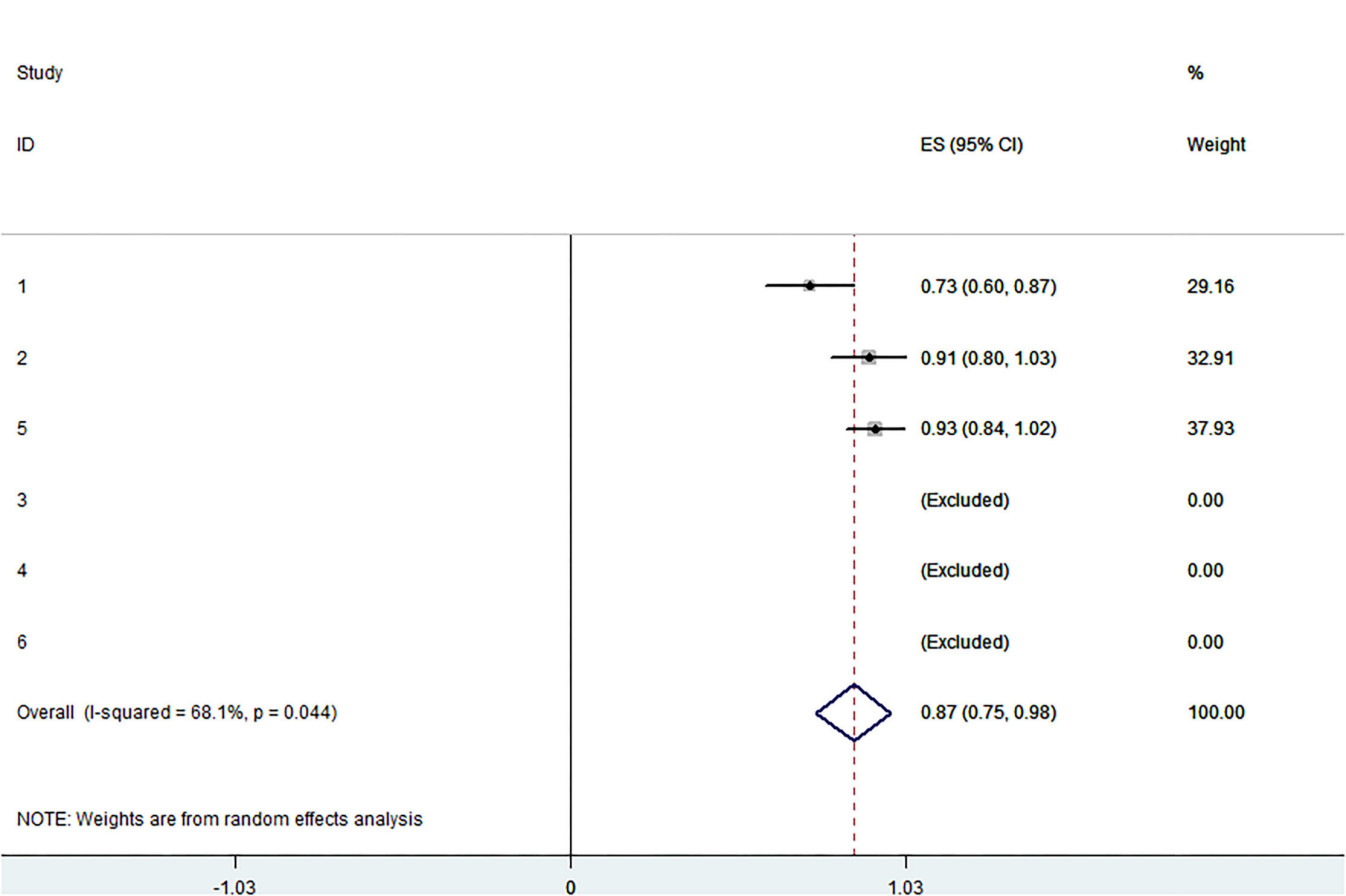
Forest plot presenting the meta-analysis of technical success rate. CI, Confidence intervals; ES, Effect size.

**Figure 5:**
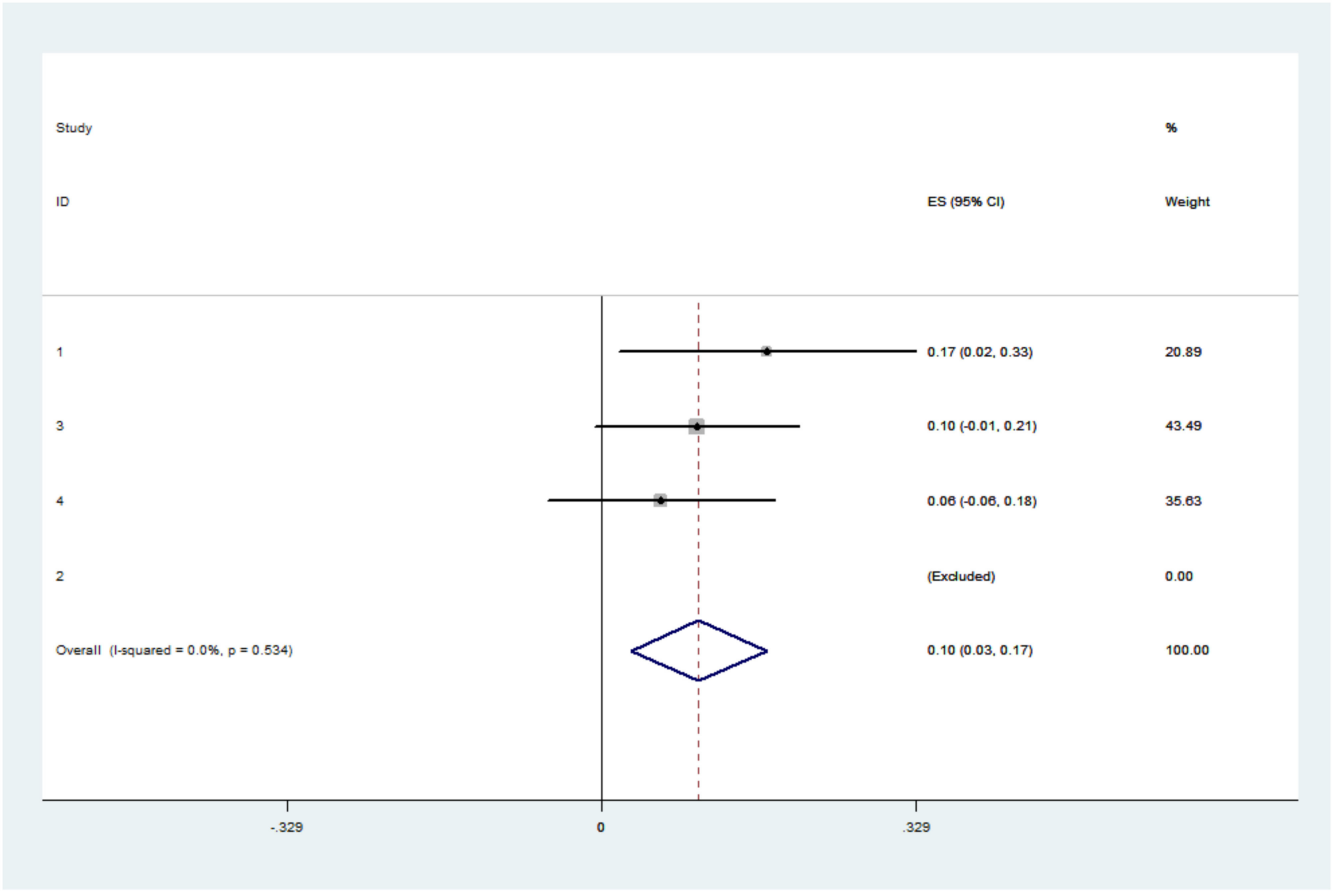
Forest plot presenting the meta-analysis of early re-intervention rate. CI, Confidence intervals; ES, Effect size.

The pooled application rate of type A was 48% (95% CI, 29–67%) (Figure 2), and pooled application rate of type B was 30% (95% CI, 16–44%) (Figure 3). The pooled technical success rate was 87% (95% CI, 75–98%) (Figure 4). Early re-intervention rate was 10% (95% CI, 3–17%) (Figure 5). Early renal infarct rate was 15% (95% CI, 8–22%). Early occlusion rate of a fenestrated renal vessel was 2% (95% CI, −1 to 4%). Midterm death rate (after 30 days) was 15% (95% CI, 8–22%). Midterm occlusion rate of a fenestrated renal vessel (after 30 days) was 8% (95% CI, 3–14%). Midterm renal infarct rate (after 30 days) was 3% (95% CI, 0–6%). Midterm re-intervention rate (after 30 days) was 30% (95% CI, 3–57%). Midterm renal failure rate (after 30 days) was 6% (95% CI, 2–10%).

## Discussion

In the present systematic review and meta-analysis, we collected and analyzed the data on standardized, off-the-shelf stent graft (p-Branch) implantation for the treatment of AAA. The results show that type A p-Branch is more used than type B. We found a pooled success rate of 87% with a lower pooled early re-intervention rate and midterm renal infarct rate of 10 and 3%, respectively.

Specifications and characteristics of the stent-graft for type A and type B have been described in detail in previous studies (16, 23). In brief, it is a tubular stent-graft with a scallop for the celiac artery consisting of one 8-mm superior mesenteric artery (SMA) and two renal artery pivot fenestrations (p-Branches). Differences in the position of the p-Branches relative to the renal artery pivotal fenestration at the origin of the SMA led to two types of p-Branches. In type A, the two branches are at the same level, while in type B, the longitudinal position of the two branches is staggered, and the left renal pivot fenestration is 4 mm lower than the right renal pivot fenestration (20). This study on p-Branch also indicated that type A was available in 54% of patients and type B was available in 49%, which is similar to our results (20). In addition, one study suggested that “OST” devices were suitable for 50–80% of patients anatomically and another study showed p-Branch stent graft was not able to incorporate visceral arteries in 40% of patients (24, 25).

The pooled technical success rate was 87%. This success rate was satisfactory; it was lower than that of off-the-shelf stents in other studies (11). Juan et al. evaluated 11 of 41 patients who were unsuitable for this stent-graft (15). Farber et al. observed 2 failures, one due to difficulty in cannulating the renal arteries and the other due to the inability to place a renal stent (19). Sveinsson et al. suggested that technical failure occurred in two emergency ruptured AAA cases where renal arteries were left unstented (16). Vessel access anatomy may also play a role in technical failure cases. Unfortunately, due to data limitations, we cannot analyze the success rates of the two types of p-Branches separately. The technical success rate of the emergent population (91.7%) is similar to that of the elective population (92.6%), which also requires more data to confirm.

The results of follow-up showed that p-Branch is not only safe and effective in selected patient populations, but also can be used in emergency situations (16). More research is needed to see if p-Branch works differently in selective and emergency settings. In addition, three studies have shown reasons for re-intervention. There were eight early interventions, two due to type III endoleak, one due to type Ic endoleak, one due to limb occlusion, one due to completion of the primary intervention, one due to SMA occlusion, one due to left renal artery occlusion, and one due to observed lower extremity ischemia. Furthermore, there were twelve late interventions, one due to type Ib endoleak, one due to type III endoleak, one due to device migration, one due to left lower extremity claudication and left femoral artery stenosis, one due to left renal stent kink, one due to hip and buttock claudication, two due to limb stenosis, three due to renal artery occlusion, three due to type II endoleak, four due to SMA stenosis, and five due to renal artery stenosis (16, 19, 20). Interestingly, there was no early intervention in the elective patient population, but there were different reasons for early and late interventions between the elective and the emergent population (16).

In our results, there was no death event in three studies. One systematic review compared the safety and efficacy of off-the-shelf fenestrated/branched grafts and physician-modified stent-grafts for the treatment of complex AAA also showed that no death at 30 days in the OTS group (11). There were 8 midterm death events, but all deaths were related to the device and procedure. Hence, the safety of the device is satisfactory.

Chuter et al. compared the results of multibranched custom-made stent-grafts with standard stent-grafts for repairing aortic aneurysms. Their results showed no significant differences in branching morphology and perioperative outcomes between the two groups, and “OTS” standard stents expanded the treatment population due to delays in the absence of manufacturing (26). However, “OST” devices may be difficult to implant due to their relative mismatch with the aortic anatomy compared to the customized stents (25). In addition to implantation problems, “OTS” devices have a higher risk of complications, such as loss of target vessels, endoleaks, and the need for open surgery, due to their lower matching with the native aortic anatomy than custom-made devices (27). In addition, branch instability can also cause issues (28).

As a study pointed out, the durability of fenestructions and branches depends on the patency of the target vessels and renal impairment (11). Early and midterm renal infarct rate and occlusion rate of a fenestrated renal vessel were low. More studies and patients are needed to confirm it strongly. There are also some limitations in this review. Firstly, the small number of published studies and patients included in the analysis as well as the high levels of heterogeneity between the included studies limit the quality of the results. Secondly, we did not include unpublished studies, such as the gray literature. Thirdly, three studies were funded by Cook Medical, which might potentially have an impact on the results.

## Conclusion

Our results showed an acceptable technical success rate of p-Branch stent graft implantation with re-intervention rate and renal failure rate that cannot be ignored. Considering these, p-Branch is a promising technology for the repair of emergent AAA, but for selective cases, it is an option that needs careful preoperative evaluation.

## Data Availability Statement

The original contributions presented in the study are included in the article/supplementary material, further inquiries can be directed to the corresponding author.

## Author Contributions

HB and HW designed experiments, performed data analysis, wrote, and revised the manuscript. LZ, ML, SW, and CZ compiled data. HB obtained funding. All authors contributed to the article and approved the submitted version.

## Funding

This study was funded by the National Natural Science Foundation of China to Hualong Bai (Grant No: 81870369), Health Science and Technology Innovation Fund for Distinguished Young Scholars of Henan Province (YXKC2021040), and Key projects of Medical Science and Technology in Henan Province to HB (Grant No: SBGJ202002035).

## Conflict of Interest

The authors declare that the research was conducted in the absence of any commercial or financial relationships that could be construed as a potential conflict of interest.

## Publisher's Note

All claims expressed in this article are solely those of the authors and do not necessarily represent those of their affiliated organizations, or those of the publisher, the editors and the reviewers. Any product that may be evaluated in this article, or claim that may be made by its manufacturer, is not guaranteed or endorsed by the publisher.

## References

[1] Nordon IM Hinchliffe RJ Holt PJ Loftus IM Thompson MM. Modern treatment of juxtarenal abdominal aortic aneurysms with fenestrated endografting and open repair–a systematic review. Eur J Vasc Endovasc Surg. (2009) 38:35–41. 10.1016/j.ejvs.2009.02.012 19346140

[2] Rao R Lane TR Franklin IJ Davies AH. Open repair vs. fenestrated endovascular aneurysm repair of juxtarenal aneurysms. J Vasc Surg. (2015) 61:242–55. 10.1016/j.jvs.2014.08.068 25240242

[3] Jones AD Waduud MA Walker P Stocken D Bailey MA Scott DJA. Meta-analysis of fenestrated endovascular aneurysm repair vs. open surgical repair of juxtarenal abdominal aortic aneurysms over the last 10 years. BJS open. (2019) 3:572–84. 10.1002/bjs5.50178 31592091 PMC6773647

[4] Cross J Gurusamy K Gadhvi V Simring D Harris P Ivancev K . Fenestrated endovascular aneurysm repair. Br J Surg. (2012) 99:152–9. 10.1002/bjs.7804 22183704

[5] Oderich GS Ribeiro M Hofer J Wigham J Cha S Chini J . Prospective, nonrandomized study to evaluate endovascular repair of pararenal and thoracoabdominal aortic aneurysms using fenestrated-branched endografts based on supraceliac sealing zones. J Vasc Surg. (2017) 65:1249–59.e10. 10.1016/j.jvs.2016.09.038 27986479

[6] Greenberg RK Sternbergh 3rd WC Makaroun M Ohki T Chuter T Bharadwaj P . Intermediate results of a United States multicenter trial of fenestrated endograft repair for juxtarenal abdominal aortic aneurysms. J Vasc Surg. (2009) 50:730–7.e1. 10.1016/j.jvs.2009.05.051 19786236

[7] Amiot S Haulon S Becquemin JP Magnan PE Lermusiaux P Goueffic Y . Fenestrated endovascular grafting: the French multicentre experience. Eur J Vasc Endovasc Surg. (2010) 39:537–44. 10.1016/j.ejvs.2009.12.008 20093051

[8] Katsargyris A Uthayakumar V Marques de Marino P Botos B Verhoeven EL. Aneurysm rupture and mortality during the waiting time for a customised fenestrated/branched stent graft in complex endovascular aortic repair. Eur J Vasc Endovasc Surg. (2020) 60:44–8. 10.1016/j.ejvs.2020.03.003 32245614

[9] Gallitto E Faggioli G Spath P Pini R Mascoli C Ancetti S . The risk of aneurysm rupture and target visceral vessel occlusion during the lead period of custom-made fenestrated/branched endograft. J Vasc Surg. (2020) 72:16–24. 10.1016/j.jvs.2019.08.273 32063442

[10] Starnes BW Heneghan RE Tatum B. Midterm results from a physician-sponsored investigational device exemption clinical trial evaluating physician-modified endovascular grafts for the treatment of juxtarenal aortic aneurysms. J Vasc Surg. (2017) 65:294–302. 10.1016/j.jvs.2016.07.123 27687323

[11] Georgiadis GS van Herwaarden JA Antoniou GA Hazenberg CE Giannoukas AD Lazarides MK . Systematic review of off-the-shelf or physician-modified fenestrated and branched endografts. J Endovasc Ther. (2016) 23:98–109. 10.1177/1526602815611887 26496957

[12] Starnes BW Tatum B Singh N. Procedural and perioperative results in patients treated with fenestrated endovascular aneurysm repair planned by automated software in a physician-sponsored investigational device exemption trial of physician-modified endografts. J Vasc Surg. (2018) 68:1297–307. 10.1016/j.jvs.2018.02.045 29706473

[13] Moher D Liberati A Tetzlaff J Altman DG. Preferred reporting items for systematic reviews and meta-analyses: the PRISMA statement. PLoS Med. (2009) 6:e1000097. 10.1371/journal.pmed.1000097 19621072 PMC2707599

[14] Konstantinou N Antonopoulos CN Jerkku T Banafsche R Kölbel T Fiorucci B . Systematic review and meta-analysis of published studies on endovascular repair of thoracoabdominal aortic aneurysms with the t-Branch off-the-shelf multibranched endograft. J Vasc Surg. (2020) 72:716–25.e1. 10.1016/j.jvs.2020.01.049. 32247700

[15] Bargay-Juan P Gómez-Palonés FJ Pepén-Moquete LA Plaza-Martínez Á Zaragozá-García JM Morales-Gisbert SM. Applicability of zenith p-Branch standard fenestrated endograft in our series. Ann Vasc Surg. (2016) 33:187–93. 10.1016/j.avsg.2015.09.030 26965825

[16] Sveinsson M Sonesson B Dias N Björses K Kristmundsson T Resch T. Five year results of off the shelf fenestrated endografts for elective and emergency repair of juxtarenal abdominal aortic aneurysm. Eur J Vasc Endovasc Surg. (2021) 61:550–8. 10.1016/j.ejvs.2020.12.012 33455820

[17] Farber MA Vallabhaneni R Marston WA. “Off-the-shelf” devices for complex aortic aneurysm repair. J Vasc Surg. (2014) 60:579–84. 10.1016/j.jvs.2014.03.258 24797555

[18] Farber MA Eagleton MJ Mastracci TM McKinsey JF Vallabhaneni R Sonesson B . Results from multiple prospective single-center clinical trials of the off-the-shelf p-Branch fenestrated stent graft. J Vasc Surg. (2017) 66:982–90. 10.1016/j.jvs.2017.01.068 28559176

[19] Farber MA Oderich GS Timaran C Sanchez LA Dawson Z. Results from a prospective multicenter feasibility study of Zenith p-Branch stent graft. J Vasc Surg. (2019) 70:1409–18.e3. 10.1016/j.jvs.2019.03.026 31255472

[20] Kitagawa A Greenberg RK Eagleton MJ Mastracci TM. Zenith p-Branch standard fenestrated endovascular graft for juxtarenal abdominal aortic aneurysms. J Vasc Surg. (2013) 58:291–300. 10.1016/j.jvs.2012.12.087 23611709

[21] Ou J Cheng SW Chan YC. The compatibility of p-branch “off-the-shelf” fenestrated endovascular graft in Asian patients with juxtarenal aortic aneurysm. J Vasc Surg. (2015) 61:1417–23. 10.1016/j.jvs.2014.12.061 25704410

[22] Guo B Moga C Harstall C Schopflocher D. A principal component analysis is conducted for a case series quality appraisal checklist. J Clin Epidemiol. (2016) 69:199–207.e2. 10.1016/j.jclinepi.2015.07.010 26307459

[23] Kristmundsson T Sveinsson M Björses K Törnqvist P Dias N. Suitability of the zenith p-Branch standard fenestrated endovascular graft for treatment of ruptured abdominal aortic aneurysms. J Endovasc Ther. (2015) 22:760–4. 10.1177/1526602815601096 26265723

[24] Kapetanios D Stana J Prendes CF Stavroulakis K Kölbel T Rantner B . Acute complex endovascular aortic repair - off-the-shelf vs. surgeon-modified stent grafts. Zentralblatt fur Chirurgie. (2021) 146:521–7. 10.1055/a-1647-3549 34666365

[25] Mendes BC Oderich GS Macedo TA Pereira AA Cha S Duncan AA . Anatomic feasibility of off-the-shelf fenestrated stent grafts to treat juxtarenal and pararenal abdominal aortic aneurysms. J Vasc Surg. (2014) 60:839–47. 10.1016/j.jvs.2014.04.038 24998837

[26] Chuter TA Hiramoto JS Park KH Reilly LM. The transition from custom-made to standardized multibranched thoracoabdominal aortic stent grafts. J Vasc Surg. (2011) 54:660–7. 10.1016/j.jvs.2011.03.005 21788114

[27] Linsen MA Jongkind V Nio D Hoksbergen AW Wisselink W. Pararenal aortic aneurysm repair using fenestrated endografts. J Vasc Surg. (2012) 56:238–46. 10.1016/j.jvs.2011.10.092 22264696

[28] Ricotta JJ 2nd Tsilimparis N. Surgeon-modified fenestrated-branched stent grafts to treat emergently ruptured and symptomatic complex aortic aneurysms in high-risk patients. J Vasc Surg. (2012) 56:1535–42. 10.1016/j.jvs.2012.05.096 22960024

